# Vascular Complications in Patients with ECMO Support after Cardiac Surgery

**DOI:** 10.3390/jcm13175055

**Published:** 2024-08-26

**Authors:** Cagdas Baran, Evren Ozcinar, Ahmet Kayan, Mehmet Cahit Saricaoglu, Ali Ihsan Hasde, Canan Soykan Baran, Ahmet Ruchan Akar, Sadik Eryilmaz

**Affiliations:** 1 Department of Cardiovascular Surgery, Heart Center, Cebeci Hospitals, Ankara University School of Medicine, 06230 Ankara, Turkey; cagdasbaran@gmail.com (C.B.); cahitsarica@gmail.com (M.C.S.); ahasde@gmail.com (A.I.H.); akarruchan@gmail.com (A.R.A.); sadik.eryilmaz@gmail.com (S.E.); 2 Department of Cardiovascular Surgery, Kirikkale High Specialization Hospital, 71300 Kirikkale, Turkey; dr.ahmet.kayan@gmail.com; 3 Department of Cardiovascular Surgery, Ankara 29 Mayıs Hospital, 06105 Ankara, Turkey; canansykn@hotmail.com

**Keywords:** extracorporeal membrane oxygenation, cardiac surgery, peripheral vascular diseases

## Abstract

**Background:** This study assessed vascular complications in patients who received extracorporeal membrane support following cardiac surgery. **Methods:** We included 84 post-cardiotomy patients who underwent extracorporeal membrane oxygenation (ECMO) from July 2018 to May 2022. Only patients connected to VA-ECMO (Veno-Arterial) via peripheral cannulation were included in this study. Vascular complications were compared between those who had ECMO placed using the percutaneous technique (*n* = 52) and those who had it placed via femoral incision (*n* = 32). **Results:** The incidence of vascular thromboembolism was significantly higher in the percutaneous technique group compared with the open technique group (*p* < 0.05). Hematomas were also more frequent in the percutaneous technique group (*p* = 0.04). Conversely, bleeding and leakage were significantly more frequent in the open technique group (*p* = 0.04). There were no significant differences between the two groups in terms of wound infections or revisions in the inguinal area following ECMO removal. The mortality rate associated with vascular ischemia was 81.2%, while the overall in-hospital mortality rate was 60.7%. **Conclusions:** The open technique for ECMO placement may reduce the risk of thromboembolic events and hematomas compared to the percutaneous technique. However, it may be associated with a higher incidence of bleeding and leakage. Both techniques show similar outcomes in terms of overall mortality and wound infections.

## 1. Introduction

Extracorporeal membrane oxygenation (ECMO) has been increasingly used for the treatment of critical patients with cardiopulmonary insufficiency [1,2,3]. Approximately 1% of patients undergoing cardiac surgery require veno-arterial (VA) ECMO due to refractory cardiopulmonary dysfunction [2,4,5]. ECMO can lead to serious consequences for patients with cardiac, pulmonary, neurological, hemorrhagic, and vascular complications [6,7]. The most commonly used route for VA-ECMO in adult patients is the percutaneous femoral artery and vein due to accessibility [8,9]. However, vascular complications associated with femoral cannulation are among the most common and serious complications of ECMO [10,11,12,13]. A distal perfusion catheter (DPC) is frequently deployed as a measure against leg ischemia [14]. The relationship between major vascular complications and ECMO patients remains unclear [15].

Studies have shown that peripherally applied VA-ECMO in cardiovascular patients should be specifically examined for vascular complications, and further studies should be conducted [14].

This retrospective study was conducted to compare complication rates and overall survival in a series of patients who received peripheral VA-ECMO via a surgical or a total percutaneous approach.

## 2. Material and Methods

### 2.1. Patients

**Figure 1:**
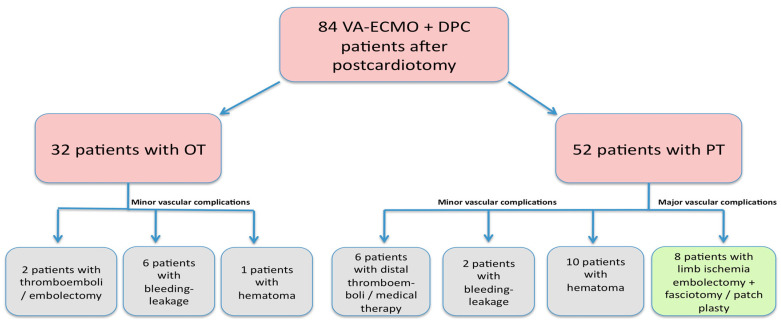
Flowchart of vascular complications. VA-ECMO: veno-arterial extracorporeal membrane oxygenation, DPC: distal perfusion catheter, OT: open technique, PT: percutaneous technique.

We retrospectively analyzed the results of a total of 84 patients who underwent VA-ECMO immediately after cardiac surgery between July 2018 and May 2022 in our clinic (Figure 1). Written consent forms were obtained from the patients before the operation, which included surgical risks/complications and information about the data that could be used in scientific studies afterward. Patients younger than 18 years were excluded from the study. The study was conducted according to the guidelines of the Declaration of Helsinki, and the research ethics board at Ankara University approved it (date: 31 July 2024, no: 2024/469).

In the study, ECMO support was provided due to refractory cardiopulmonary insufficiency developing in the weaning phase of CPB (cardiopulmonary bypass). Peripheral cannulation was used as the interventional route in these patients. Femoral artery and venous cannulation were performed percutaneously in 52 patients using a Doppler ultrasonography (USG). Thirty-two patients underwent femoral exploration, and ECMO cannulas were placed in the femoral artery and vein. All patients had DPCs placed. The study end-points were the difference in survival and vascular complications, acute limb ischemia, bleeding, compartment syndrome, thromboembolism, and infection.

### 2.2. ECMO Cannulation

The ECMO circuit utilized included a centrifugal pump (Jostra Rotaflow; Maquet Cardiopulmonary, Rastatt, Germany) and an oxygenator (Jostra Quadrox; Maquet Cardiopulmonary, Rastatt, Germany). The prime solution for the perfusion lines consisted of Ringer’s Lactate with an initial addition of 1000 IU/L of heparin. The technique used for peripheral cannulation was based on the surgeon’s preference. All ECMOs were placed in the operation room. The common femoral artery (CFA) and vein (usually right) were preferred in all patients.

For the choice of the percutaneous technique (PT), the CPB procedures were completed, but the decision to urgently place VA-ECMO was made due to limitations in hemodynamics and cardiac function despite high-dose inotropic drugs. For the choice of the open surgical technique (OT), the group failed to wean from the CPB and came out of the bypass with the support of ECMO. For both techniques, 12 Fr DPCs were placed antegrade into the proximal superficial femoral artery (SFA).

### 2.3. Percutaneous Technique (PT)

**Figure 2:**
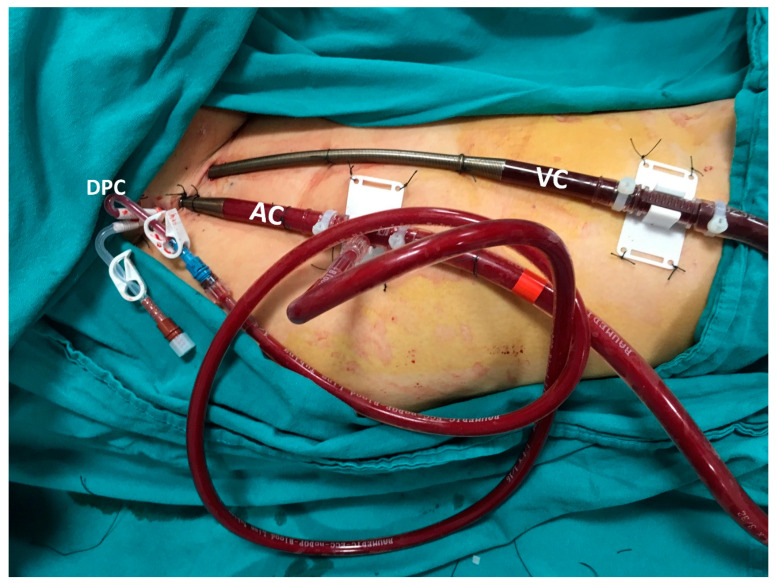
Percutaneous technique for VA-ECMO. AC: arterial cannula, VC: venous cannula, DPC: distal perfusion catheter.

The femoral artery and vein of the patient were evaluated in detail by Doppler USG. Atherosclerosis, thrombus formation, vessel diameter, and the flow patterns of the CFA were determined with Duplex scanning. After the Seldinger technique was used with US-guided femoral vein puncture, a flexible J-tip guidewire was advanced from the femoral vein. A 21 or 23 Fr venous cannula (HLS Cannulae, Maquet^®^, Rastatt, Germany) was inserted over the wire to the cavo-atrial junction under transesophageal echocardiography (TEE) guidance. Using the same method, the CFA 17 to 19 Fr/15 cm arterial cannula (Maquet, Rastatt, Germany) were placed (Figure 2).

### 2.4. Open Technique (OT)

**Figure 3:**
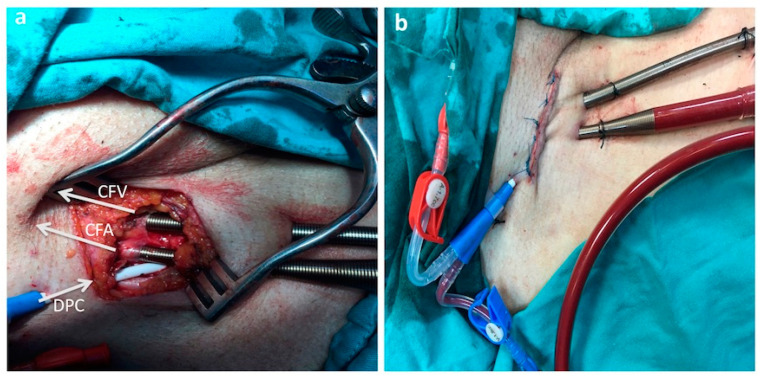
Open technique for VA-ECMO. CFA: common femoral artery, CFV: common femoral vein, DPC: distal perfusion catheter, (**a**): DPC and veno-arterial cannulas were placed near the incision. (**b**): Mini-incision was closed after the cannula fixation.

After the femoral region was assessed with Doppler USG, the femoral artery and vein were explored with a mini femoral incision. A venous cannula (21–23 Fr/55 cm) (Maquet, Rastatt, Germany) and an arterial cannula (17–19 Fr/15 cm) (Maquet, Rastatt, Germany) were placed away from the plaque structures using the Seldinger technique near the incision (Figure 3a). The mini-incision was sutured after the fixation procedure was completed (Figure 3b).

### 2.5. Decannulation Technique

ECMO decannulation was performed in the operating room under local anesthesia and sedation in patients who survived and successfully managed ECMO weaning.

Decannulation procedure in patients with OT: Sutures were easily removed and the femoral artery and vein were accessed. The femoral artery was turned proximally and a purse suture was placed around the cannula with 5.0 prolene. The ECMO cannulas were clamped, the femoral artery cannula was pulled, and the prolene was ligated. Placing a prolene suture around the incision site also decannulated the femoral vein. A U suture of 5.0 prolene was placed around the DPC and then removed.

Decannulation procedure in patients with PT: A longitudinal incision was made to keep the femoral artery cannula in the middle, and the tissues and hematomas, if present, were explored. The femoral artery was proximally suspended with a silastic tape. If vascular repair was to be performed, a proximal and distal femoral arterial vascular clamp was placed immediately after decannulation. Otherwise, a 5.0 prolene purse suture was placed, and decannulation was performed. The femoral venous cannula and DPC were decannulated using a similar technique.

With the PT, the decannulation procedure involves specific challenges that can impact complication rates, including wound infection and hematoma.

This approach provides access to inspect and manage any hematomas or thrombus that may be present.

During this process, hematomas are thoroughly examined, and any necessary vascular repairs are performed to address bleeding sources. This thorough exploration is crucial for ensuring complete hemostasis and managing complications.

Hematoma Management and Additional Procedures:

The frequent observation of hematomas with the PT necessitates careful wound management. Hematomas can increase the risk of bleeding and may complicate the healing process.

Additional procedures, such as suturing and careful wound closure, are employed to minimize bleeding and ensure proper wound healing. The need for these procedures can contribute to a higher incidence of wound-related complications with the PT.

By contrast, the OT benefits from a more direct and controlled approach to decannulation.

The OT allows for the direct visualization and management of the femoral vessels through a mini incision. This direct approach facilitates the precise placement and removal of cannulas and enables better control of bleeding.

The visibility and access provided by the OT typically result in fewer complications related to hematomas, as the vessels are inspected and managed more effectively.

The open technique generally results in fewer issues with hematoma formation, as the direct approach allows for better control and monitoring of the decannulation site. This approach often leads to more straightforward wound management and fewer postoperative complications.

The detailed exploration and additional procedures during decannulation with the PT group, such as hematoma management and vessel inspection, are essential for addressing potential complications. These factors contribute to the observed similarities in wound infection and bleeding risks between the PT and OT. The thorough exploration and management required for the PT, while necessary, may result in complications that are comparable to those seen with the OT.

### 2.6. Statistical Analysis

Descriptive statistics are presented as frequencies and percentages, means ± standard deviations, and medians (with minimum and maximum values), as applicable. Differences between the two groups for continuous variables were assessed using Student’s *t*-test. For ordinal or non-normally distributed continuous variables, the Mann–Whitney U test was employed. Categorical variables were analyzed using the Chi-square test or Fisher’s Exact test, as appropriate. All statistical tests were two-tailed, with a significance level set at a *p*-value of less than 0.05. The Kaplan–Meier method was utilized to estimate cumulative survival and create survival curves, while the log-rank (Mantel–Cox) test was applied to compare the differences between these curves. All statistical analyses were performed using SPSS version 23.0 (IBM, Chicago, IL, USA).

## 3. Results

The demographic and preoperative data of the patients are summarized in Table 1. Sixteen of the patients included in the study (19%) required a redo of the operation. Of the patients requiring VA-ECMO after postcardiotomy, 14 (16%) had isolated coronary artery bypass grafting, 42 (50%) had coronary bypass and valve replacement/repair, 26 (31%) had multiple valve operation, and 2 (3%) underwent Bentall + Hemiarch operation.

The intraoperative data of these patients, which constitute high-risk groups, are given in Table 2. Because of the refractory cardiogenic shock developed after the postcardiotomy, VA-ECMO support was provided urgently to all patients. These patients showed signs of low cardiac output syndrome despite maximum doses of dopamine, Dobutrex, noradrenaline, and adrenaline infusions (mean arterial blood pressure ≤ 50 mmHg, severe contraction defect in transesophageal echo, abnormal blood gas values). All patients were monitored at a flow rate of 4–6 L/minute.

Heparin infusion was started and activated clotting time was measured every 6 h with a target activated clotting time of 160–180 s. Activated partial thromboplastin time was measured twice daily with a target of 50–60 s.

**Figure 4:**
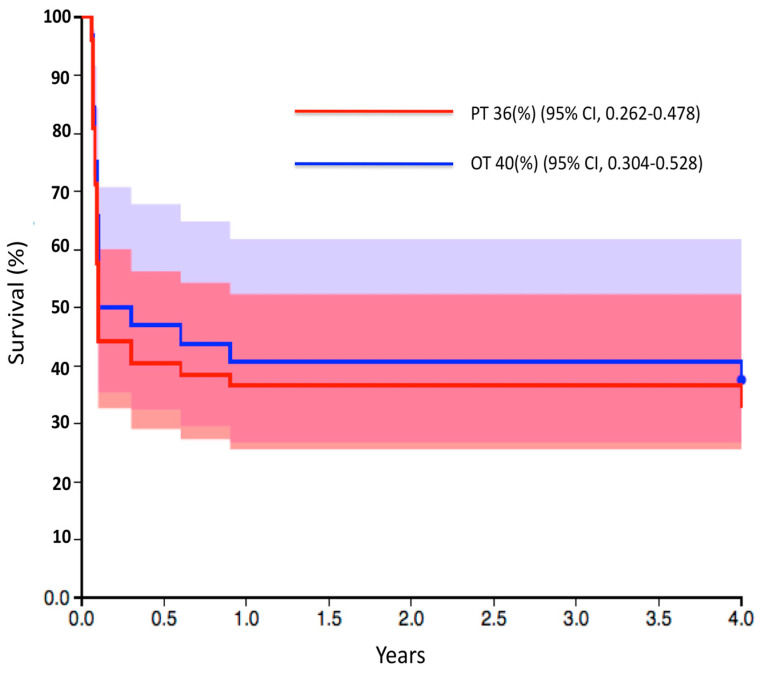
Kaplan–Meier survival curves showing cumulative survival of patients. The differences between the VA-ECMO perfusion techniques were non-significant (*p* = 0.62). CI: confidence interval, OT: open technique, PT: percutaneous technique; *p*-values were determined with the use of the Mantel–Cox log-rank test.

In the intensive care unit follow-up, embolectomy and fasciotomy were performed due to ischemia in four of the PT-treated patients. Digital necrosis developed in six patients due to distal thromboembolism. Also in this group, four patients underwent embolectomy and saphenous vein patch-plasty operation during ECMO withdrawal (Figure 1). Thromboembolism was observed in only two patients who had ECMO placement with the OT, and embolectomy was performed during ECMO withdrawal (*p* = 0.02). Doppler USG detected hematomas as blood clots accumulating around the cannulas. Hemorrhage was evaluated as bleeding from the cannula or incision area. Hematoma development was observed in 10 patients using the PT, whereas only 1 patient had a hematoma in the OT group (*p* = 0.04). Bleeding/leakage was observed in two patients in the PT group and six patients in the OT group (*p* = 0.04). Of the surviving patients, four wound infections/revisions in the femoral region were observed in the PT group and two were observed in the OT group (*p* = 1). There were no significant differences between the groups in terms of mortality or length of hospital stay (Table 3). The mortality rate in patients with vascular ischemia was 81.2% (13 patients), while in-hospital mortality was 60.7% (51 patients). The four-year survival rate was 36% (95% CI = 0.26–0.47) in the OT group and 40% (95% CI = 0.30–0.52) in the PT group (*p* = 0.62) (Figure 4).

## 4. Discussion

Vascular complications are one of the most common complications in patients with ECMO [7]. There are limited data on the effects of vascular complications of ECMO on outcomes [13,15]. Femoral VA-ECMO-associated limb ischemia has an incidence of between 10% and 70% for both open and percutaneous cannulation [14,16]. However, the Extracorporeal Life Support Organization (ELSO) does not follow the extremity ischemia rates in particular, and therefore, the actual incidence is unknown [17]. The increased risk for ischemic complications in the ECMO population has been associated with peripheral vascular disease, possibly due to insufficient vascular circulation [9]. It has been suggested that the CFA diameter increases with age and that young patients have less distal flow around the cannula and are thus susceptible to ischemia [12]. There is evidence that having a DPC placed actually increases the perfusion of the lower extremity [18]. Tanaka et al. [19] emphasized that vascular complications that develop in patients with femoral VA-ECMO have a negative impact on surveillance and that DPC reduces the risk of vascular complications. In this study, according to multiple variance analysis, the only important predisposing factor for vascular complications was the absence of a DPC, and ischemic complications affected the outcome more than bleeding complications [19]. In another study, prophylactic antegrade DPC was reported to reduce ischemia complications in the leg [20]. We use routine DPC in our patients, as we are a clinic with an ECMO center.

It was emphasized that there was no correlation between ECMO-related vascular complications and the increased risk of in-hospital mortality in adult patients [21]. A recent study reported that the use of ECMO in patients with cardiogenic shock was associated with high in-hospital mortality [22].

**Figure 5:**
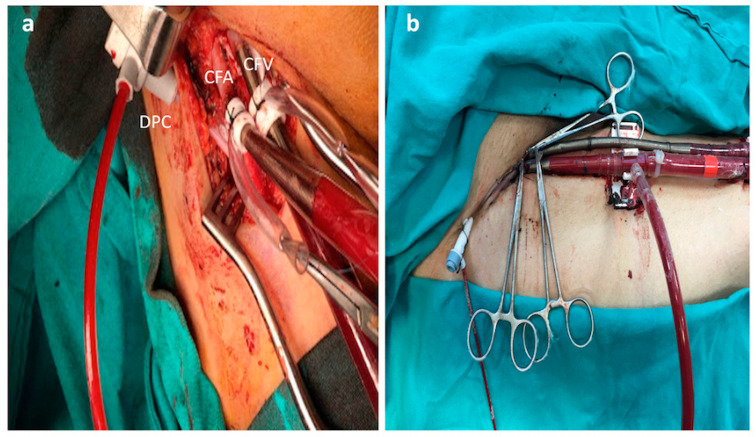
Previous open technical method. (**a**): Once the purse sutures are placed, the cannulas are placed directly over the femoral artery and vein. (**b**): The tourniquets and cannulas are fixed to the outside of the closed incision seams. CFA: common femoral artery, CFV: common femoral vein, DPC: distal perfusion catheter.

Refractory cardiopulmonary insufficiency after postcardiotomy is a condition that requires urgent ECMO support. Another article emphasized the need for VA-ECMO support in patients with refractory cardiogenic shock [23]. The fact that the equipment is already on the operating table makes it easier to set up the ECMO with the open technique. Although the open technique method helps us evaluate the femoral artery with direct eye and palpation, it clearly determines our target for the placement of the catheter and cannula. We can advance the guidewire more safely. Furthermore, the OT method allows us to reach the transport and maneuver more easily when the ECMO withdrawal stage is reached. In cannulas placed with PT, the frequently observed hematoma and the entry level may be at higher levels toward the iliac artery, which may make transportation and manipulation more difficult. In our initial open technical experience, we performed a direct puncture of the femoral artery and vein for inserting the cannulas and DPC into the purse sutures. The tourniquets and cannulas were fixed to the outside of the closed incision seams (Figure 5a,b). However, this method was abandoned due to the high bleeding/leakage rates, and the cannulas were fixed by entering through the skin area near the incision line (Figure 3).

Despite DPC placement, we believe that thromboembolic events and extremity ischemia in the PT group, reduced vascular dominance, recurrent frequent punctures during the operation, and unstable plaque structures separating from the vessel wall during DPC placement contribute to complications.

## 5. Conclusions

Vascular complications are a significant concern in ECMO therapy. Our study reveals that the percutaneous technique (PT) for ECMO placement is associated with a higher incidence of thromboembolic events and hematomas compared with the open technique (OT). Conversely, the open technique is linked to a greater frequency of bleeding and leakage complications. While both techniques resulted in similar rates of wound infections and overall mortality, the open technique appears to offer a benefit in reducing thromboembolic complications. Therefore, the choice of technique may be guided by balancing the risks of thromboembolic events and bleeding, tailored to individual patient circumstances.

## Figures and Tables

**Table 1:** Patient demographic data.

Baseline Characteristics	OT VA-ECMO (*n* = 32)	PT VA-ECMO (*n* = 52)	*p* Value
Age, years, mean ± SD	55 ± 14.4	58 ± 16.6	0.4
Male gender, *n* (%)	22 (68.7)	38 (73)	0.4
BMI (kg/m^2^), mean ± SD	25.0 ± 3.5	25.2 ± 3.4	0.6
Hypertension, *n* (%)	26 (81.2)	42 (80.7)	0.7
Hyperlipidemia, *n* (%)	21 (65.6)	34 (65.3)	0.8
Smoke, *n* (%)	14 (43.7)	22 (42.3)	1
Diabetes mellitus, *n* (%)	15 (46.8)	24 (46.1)	1
Dialysis requirement, *n* (%)	1 (3)	3 (5.7)	1
COPD, *n* (%)	8 (25)	14 (27)	1
LVEF, mean ± SD	30 ± 8.4	30 ± 6.2	1
Preoperative inotropic agents, *n* (%)	1 (3)	2 (3.8)	1
Preoperative IABP support, *n* (%)	1 (3)	2 (3.8)	1
Urgent cardiac operation, *n* (%)	4 (12.5)	7 (13.4)	1
Redo operation, *n* (%)	5 (15.6)	11 (21.1)	0.5
Peripheral artery disease, *n* (%)	1 (3)	1 (3)	1
Euroscore II (high-risk, >6), *n* (%)	28 (87.5)	46 (88.4)	1

Data are presented as *n* (%) or mean ± standard deviation. OT: open technique, PT: percutaneous technique, VA-ECMO: veno-arterial extracorporeal membrane oxygenator, BMI: body mass index, COPD: chronic obstructive pulmonary disease, LVEF: left ventricular ejection fraction, IABP: intra-aortic balloon pump.

**Table 2:** Operative data of patients undergoing VA ECMO.

Variables	OT VA-ECMO (*n* = 32)	PT VA-ECMO (*n* = 52)	*p*-Value
CABG, *n* (%)	6	8	0.7
CABG + Valve repair/replacement, *n* (%)	15	27	0.8
MVR + AVR + TRA/TVR, *n* (%)	10	16	1
Bentall + Hemiarch, *n* (%)	1	1	1
CPB time (min), mean ± SD	145 ± 24	154 ± 38	0.2
X clamp time (min), mean ± SD	80 ± 12	84 ± 11	0.1
Postoperative inotropic support, *n* (%)	32	52	1
Lactate > 10 (0.5–1.6 mmol/L), *n* (%)	24	42	0.5
PH < 7.35 (7.35–7.45 U), *n* (%)	19	24	0.2

Data presented as *n* (%) or mean ± standard deviation. OT: open technique, PT: percutaneous technique, VA-ECMO: veno-arterial extracorporeal membrane oxygenator, CABG: coronary artery bypass grafting, MVR: mitral valve replacement, AVR: aortic valve replacement, TRA: tricuspid ring annuloplasty, TVR: tricuspid valve replacement, CBP: cardiopulmonary bypass.

**Table 3:** Vascular complications and postoperative data during VA-ECMO support.

Variables	OT VA-ECMO (*n* = 32)	PT VA-ECMO (*n* = 52)	*p* Value
Ipsilateral limb emboli-ischemia, *n* (%)	2 (6.2)	14 (26)	0.02
Hematoma, *n* (%)	1 (3.1)	10 (19)	0.04
Bleeding/leakage, *n* (%)	6 (18)	2 (3.8)	0.04
Superficial wound infection/revision, *n* (%)	2 (6.2)	4 (7.6)	1
VA-ECMO run (days), mean ± SD	12.4 ± 2.8	13.6 ± 3.4	0.09
ICU stay (days), mean ± SD	18.2 ± 3.7	19.4 ± 4.1	0.1
Hospital stay (days), mean ± SD	32 ± 5.4	30 ± 6.2	0.1
In-hospital mortality, *n* (%)	18 (56.2)	33 (63.4)	0.6

Data are presented as *n* (%) or mean ± standard deviation. OT: open technique, PT: percutaneous technique, VA-ECMO: veno-arterial extracorporeal membrane oxygenator, ICU: intensive care unit.

## Data Availability

Data are contained within the article.
